# Measuring progress with clinical governance development in New Zealand: perceptions of senior doctors in 2010 and 2012

**DOI:** 10.1186/s12913-014-0547-8

**Published:** 2014-11-04

**Authors:** Robin Gauld, Simon Horsburgh

**Affiliations:** Centre for Health Systems, Department of Preventive and Social Medicine, Dunedin School of Medicine, University of Otago, PO Box 56, Dunedin, 9054 New Zealand

**Keywords:** Clinical Governance, Hospital Specialists, Survey, New Zealand

## Abstract

**Background:**

Clinical governance has become a core component of health policy and services management in many countries in recent years. Yet tools for measuring its development are limited. We therefore created the Clinical Governance Development Index (CGDI), aimed to measure implementation of expressed government policy in New Zealand.

**Methods:**

We developed a survey which was distributed in 2010 and again in 2012 to senior doctors employed in public hospitals. Responses to six survey items were weighted and combined to form the CGDI. Final scores for each of New Zealand’s District Health Boards (DHBs) were calculated to compare performances between them as well as over time between the two surveys.

**Results:**

New Zealand’s overall performance in developing clinical governance improved between the two studies from 46% in 2010 to 54% in 2012 with marked differences by DHB. Statistically significant shifts in performance were evident on all but one CGDI item.

**Conclusions:**

The CGDI is a simple yet effective method which probes aspects of organisational commitment to clinical governance, respondent participation in organisational design, quality improvement, and teamwork. It could be adapted for use in other health systems.

## Background

The concept and use of the term ‘clinical governance’ emerged in the late-1990s in the United Kingdom and has since become central to health policy in a range of countries [1–3]. Built around an arguably indistinct set of ideas, clinical governance has been defined in various ways, making it difficult for both managers and clinicians in terms of what they should be aiming for by way of structures, processes and outcomes [4]. In a general sense, and following the classic Scally and Donaldson definition [5], clinical governance involves health care professionals leading the way in quality improvement efforts, ensuring practices are evidence-based, and working to build team-based and systematised service delivery processes. Central to clinical governance is the idea that clinicians are best placed to encourage performance improvement amongst peers [6]. Clinical governance may therefore be expressed in terms of health professionals having two roles: improving the care delivery system, as well as providing care. Extracting from this, clinical governance might be seen in both organisational structures and processes, with an expectation that improved outcomes will be delivered.

An increasing volume of clinical governance research is largely focused on the United Kingdom National Health Service (NHS) and based on case study approaches. For the most part, the research reveals challenges in developing leadership models that feature genuine health professional involvement and often limited opportunities and management support for clinical governance per se [7–9]. These studies also find difficulties in getting health professionals to see governance and leadership as a ‘step up’ and an ‘important calling’ above and beyond clinical service delivery. In contrast with many other health services and management fields, there is a dearth of clinical governance research aimed at assessing development of structures and processes or tracking development over time. A 2010 study presented a method for tracking clinical governance development across a disparate range of activities, providing something of a conceptual framework for evaluation [10]. However, as far as we are aware, this has not been put to practical use. Others have sought to measure levels of ‘medical engagement’, developing proprietary measures for this [11,12]. Then there are various tools produced by government agencies for health care providers to use for self-assessment. Examples include a Western Australian tool which asks organisational leaders to rate their performance on eight dimensions of clinical governance development and provide evidence to support their assessment [13]. The Irish National Health Executive has taken a similar approach [14]. Such tools can play an important role in helping providers reflect on their efforts and performance and highlight for policy makers areas in which additional resources or incentives may be required. However, they are mostly applied at the organisational level, can be time-consuming to complete, and may not necessarily capture the perspectives of front-line health professionals. There is, therefore, a need for tools that can be used consistently and over time to compare performances amongst health care providers and provide an indication of commitment to clinical governance and its development. Such tools need to be independently-developed, easy-to-use and incorporate the perspectives of front-line professionals. There is also a need to test and validate tools through practical application.

With this in mind, in an earlier project we created the Clinical Governance Development Index (CGDI) designed for tracking clinical governance development, with a focus on structures and processes and on measuring implementation of government policy [15]. The setting was the government-dominated health system of New Zealand. This caters to a population of 4.4 million, is around 80% government-funded from general taxes with the remainder from private sources. In 2013, total health expenditure was 10.3% of GDP. The institutional arrangements that underpin New Zealand’s health system are not dissimilar to those of other government-funded jurisdictions [16,17], although being a small country with a centralised political system means that change can be rapid [18]. The public sector dominates hospital care which is provided via 20 District Health Boards. Public hospitals are free of patient charges; private hospitals offer only elective services without government subsidy, and there are no private emergency services. Primary medical care, by contrast, is largely privately provided, albeit with considerable government subsidies to offset patient co-payments.

In early-2009, and drawing heavily on material and experiences from the NHS – particularly the Leadership Qualities Framework [19], a government-commissioned working party made several recommendations for clinical governance development [20]. These included that: District Health Boards (DHBs) must establish governance structures ensuring a partnership of clinical and corporate management; these Boards and their Chief Executives must enable strong clinical leadership and decision making and promote this throughout the organisation; clinical governance must cover the whole patient journey, with decision making devolved to the appropriate level; and management must identify clinical leaders and support their development. The Minister of Health announced the working party’s recommendations were to become government policy, with implementation by DHBs an immediate priority. In 2010, we therefore sought to measure the extent to which clinical governance was being implemented by surveying public hospital medical specialists, a group finely attuned to the nuances of clinical governance and likely to be aware of changes in leadership and organisational structures. From this project, we developed the CGDI. In 2012, we conducted a follow-up study to investigate progress two years on. This article outlines the methods for the two surveys, results, derivation of the index and implications for utilising it.

## Methods

For the 2010 study, we developed a fixed-response 11-item survey, with an additional eight background questions and comments box. Survey items related directly to the key policy statements in the clinical governance working party’s report [20]. Thus, respondents were asked to rate the extent of familiarity with clinical governance concepts and policy. In a series of related questions, they rated the extent to which they perceived their employer organisation was working to develop and support clinical governance and partner with clinicians, from the level of the board and senior management through to front-line services. The survey was peer-reviewed through the development process by six researchers and medical professionals, along with the 10 members of the National Executive of the Association of Salaried Medical Specialists (ASMS), the national public hospital specialists union. A draft was then piloted among two groups of hospital specialists (22 in total) with further adjustments following feedback. Through the scrutiny of these groups, the survey questions and design fulfilled the standards of both ‘face’ and ‘construct’ validity [21].

All New Zealand public hospital specialists are salaried employees of one of the 20 District Health Boards they work for. Over 90% of these specialists are members of the ASMS. The self-completed survey was distributed in paper form by ASMS to its 3402 members in June 2010, with two follow-up reminders. In August 2010, a web-based survey sought participation with an email invite to those who had not responded to the paper version, with two subsequent email reminders. Data from paper surveys were converted into electronic form and merged with data from the web survey, with no significant differences in response patterns found.

The CGDI was formed through combining responses from seven survey items representing related aspects of processes involved in developing clinical governance. The index demonstrated good internal consistency, with a Cronbach’s alpha of 0.80.

The 2012 survey was modified, and its validity enhanced, in a consultative process that involved over 200 health professionals employed by DHBs, government policy makers and DHB managers. The 2012 study had a broader scope in that it included all health professionals. Thus, a total of 41030 professionals, including doctors, nurses, midwives and allied service providers, were invited by their DHB human resources department to participate in an online survey. Inclusion criteria were that invitees must be registered health professionals, in on-going full- or part-time employment with their DHB, and with an official DHB email address (in theory, any such DHB employee has one). Invites, containing a link to the survey website, were sent to employee email addresses. Three follow-up emails were sent to employees at weekly intervals. A national communications campaign ensured that all 19 participating DHBs distributed standard instructions (Canterbury DHB did not participate due to the earthquake recovery process).

The 2012 survey did not include one of the survey items which contributed to the CGDI in the 2010 survey, and minor changes in wording were made to some items. This was to improve their relevance to an inter-professional audience and reduce any perceived biases associated with the ASMS involvement in the earlier survey. An amended CGDI was therefore created for the 2012 survey using the remaining common six CGDI survey items. Table 1 lists the items from each version of the CGDI, as well as how the items are scored. An individual’s CGDI was arrived at by summing their scores for each item. These raw scores were converted to percentages for final presentation. ‘Don’t know’ responses were treated as missing values. A CGDI was only calculated for individuals with no missing values. To obtain the overall CGDI for a DHB, the mean CGDI of all individuals from that DHB was calculated. For the purposes of analyses for this article, only data from medical specialists in the 2012 survey were included in the comparisons (since the 2010 survey only included these professionals). All analyses were performed using the R statistical system version 2.12 [22].

**Table 1:** **Survey items from the 2010 and 2012 versions of the CGDI**

**Item**	**Scoring**	**CGDI item from 2010 survey**	**CGDI item from 2012 survey**
1	Yes = 1, No = 0	To your knowledge, has your DHB board established a governance structure that ensures a partnership between clinicians and management?	To your knowledge, has your DHB established governance structures that ensure a partnership between health professionals and management?
2	A great extent = 2, Some extent = 1, No extent = 0	To what extent are clinicians in your hospital involved in a partnership with management with shared decision making, responsibility and accountability?	To what extent are health professionals in your DHB involved in a partnership with management with shared decision making, responsibility and accountability?
3	A great extent = 2, Some extent = 1, No extent = 0	To what extent are clinicians in your hospital involved as full active participants in all governance decision making processes?	To what extent are health professionals in your DHB involved as full active participants in the design of organisational processes?
4	A great extent = 2, Some extent = 1, No extent = 0	To what extent do you believe that quality and safety is a goal of every clinical initiative in your DHB hospital?	To what extent do you believe that quality and safety is a goal of every clinical initiative in your DHB?
5	A great extent = 2, Some extent = 1, No extent = 0	To what extent do you believe that quality and safety is a goal of every administrative initiative in your DHB hospital?	To what extent do you believe that quality and safety is a goal of every clinical resourcing or support initiative in your DHB?
6	A great extent = 2, Some extent = 1, No extent = 0	To what extent has your DHB sought to give you responsibility for clinical service decision making in your clinical areas?	To what extent has your DHB sought to give responsibility to your team for clinical service decision making in your clinical areas?
7	A great extent = 2, Some extent = 1, No extent = 0	To what extent has your DHB leadership sought to identify clinical leaders?	Not included.

The study protocol and survey tool were reviewed, including for ethical considerations, and approved by the National Executive of the Association of Salaried Medical Specialists, National Executive of the National Health Board, Board and executive team of the Health Quality and Safety Commission, and CEO and leadership teams of the participating DHBs.

## Results

The 2010 survey achieved a response rate of 52%; some 32% of senior medical officers responded to the 2012 survey. Response rates differed by DHB, as may be expected of a large multi-regional study of this nature. In 2010, the response rate ranged from 39% to 70% between individual DHBs (mean = 51%, SD = 7%); in 2012, the range was 10% to 73% (mean = 39%, SD = 16%). Respondent characteristics in both surveys were compared with DHB health workforce data and found to be close to those of all potential respondents.

After applying the various exclusions outlined in the previous section, 1487 respondents from the 2010 survey and 1313 from the 2012 survey were included in the analysis. Of these, 944 (63%) and 856 (65%) had complete data and could have a CGDI score computed. The results are presented in Table 2. As represented in the increase from 46 to 54% in the mean score, the results show considerable progress across the DHBs in the two years between the surveys. Several DHBs demonstrated substantial improvement with, in one case, an increase from 44 to 64% in the index score. One DHB’s score decreased. It needs to be acknowledged that this is one of the smallest DHBs and a small number of respondents to the 2012 survey meant the score was highly sensitive to small changes in response patterns. There was evidence of substantial variation in the CGDI scores of different DHBs in both the 2010 and 2012 surveys. In 2010, the range was 38–55%; in 2012, the level of variation broadened to 36–65%.

**Table 2:** **Comparison of CGDI scores for the 2010 and 2012 surveys**

**DHB**	**2010 survey**	**2012 survey**	**Change**
South Canterbury	44%	64%	20
Whanganui	40%	58%	18
Hawke’s Bay	44%	61%	17
Taranaki	47%	64%	17
Counties Manukau	52%	65%	13
Nelson Marlborough	41%	53%	12
Waitemata	47%	58%	11
Wairarapa	39%	49%	10
Northland	46%	55%	9
Lakes	49%	58%	9
Waikato	46%	52%	5
MidCentral	43%	47%	4
Auckland	49%	53%	4
Hutt Valley	49%	52%	3
Bay of Plenty	38%	41%	2
Capital and Coast	54%	57%	2
Tairawhiti	55%	56%	1
Southern	42%	42%	0
West Coast	41%	36%	−5
Mean	46%	54%	8

Note that the numbers have been rounded, which may make the difference not appear to equal the difference between the two survey scores.

Table 3 contains the mean of the mean scores for respondents across DHBs for 2010 and 2012. The fourth column in the table lists the change in the means over the two surveys for each CGDI item, showing significant improvement in all but item 3, with the most marked improvement in items 5 and 6.

**Table 3:** **Change in mean DHB ratings (i.e. mean of the DHB means) for each of the CGDI items across the two surveys, with higher scores indicating more positive mean responses**

	**2010**	**2012**			
**Survey item (scoring range)**	**DHB Mean (SD)**	**DHB Mean (SD)**	**Change**	**95% CI for Change**	***p*** **=**
To your knowledge, has your DHB established governance structures that ensure a partnership between health professionals and management? (0–1)	0.64 (0.12)	0.71 (0.12)	+0.08	+0.03 – +0.12	0.0040*
To what extent are health professionals in your DHB involved in a partnership with management with shared decision making, responsibility and accountability? (0–2)	0.87 (0.15)	1.04 (0.16)	+0.17	+0.07 – +0.27	0.0023*
To what extent are health professionals in your DHB involved as full active participants in the design of organisational processes? (0–2)	0.88 (0.14)	0.93 (0.15)	+0.05	−0.04 – +0.14	0.2533
To what extent do you believe that quality and safety is a goal of every clinical initiative in your DHB? (0–2)	1.12 (0.09)	1.28 (0.14)	+0.16	+0.09 – +0.23	0.0001*
To what extent do you believe that quality and safety is a goal of every clinical resourcing or support initiative in your DHB? (0–2)	0.83 (0.09)	1.08 (0.14)	+0.25	+0.17 – +0.33	0.0000*
To what extent has your DHB sought to give responsibility to your team for clinical service decision making in your clinical areas? (0–2)	0.74 (0.10)	1.00 (0.19)	+0.26	+0.16 – +0.36	0.0000*

Three DHBs have been removed because of low numbers (fewer than 10) or not taking part in both surveys, leaving 924 and 849 respondents for the 2010 and 2012 surveys respectively.

SD = Standard Deviation.

95% CI = 95% Confidence Interval.

* = Statistically significant at the p = 0.05 level.

p-values obtained using Student’s *t*-test for paired data, with the DHBs across the two surveys as the pairs.

## Discussion

With our 2010 study, we developed the CGDI and, following feedback from New Zealand health care professionals, their employers and policy makers, modified the original survey tool for our 2012 follow-up. In this regard, and given the input of a wide range of individuals into the 2012 instrument design, we believe we have enhanced its validity.

We argue that the CGDI offers a straightforward and efficient means of measuring the extent to which a health care organisation is working to facilitate clinical governance. There are various reasons for this, including that the CGDI draws its measures from the perspective of health professionals. While there is an obligation on the part of professionals to take up opportunities to get involved in clinical governance activities, robust clinical governance demands managerial facilitation. Thus, professionals should be able to gauge the extent to which managerial and organisational activities are focused on building clinical governance, as well as the level of emphasis on clinical governance components such as quality and safety improvement. Importantly, the CGDI mixes items that probe both management and clinician activities; in our study, these items sought to measure the implementation of stated policy. The CGDI can also be used as a yardstick for assessing performances on clinical governance over time as well as between organisations which can be useful as a stimulant for improvement. Finally, in contrast with other tools for measuring clinical governance [10], the CGDI is focused on a defined set of important goals and processes that includes relationships between managers and health professionals, involvement in organisational design, and quality improvement which are central to the original Scally and Donaldson definition of clinical governance. This involved developing:

… a system through which health organisations are accountable for continuously improving the quality of their services and safeguarding high standards of care by creating an environment in which excellence in clinical care will flourish [5].

Our use of the CGDI suggests positive progress in clinical governance development across New Zealand’s DHBs, at least from the perspective of senior doctors. In this regard, it indicates traction with the policy implementation process, with some DHBs making considerably more progress than others. The magnitude of change in several DHBs leads to questions of how they achieved this, and why others did not. In some cases, DHBs obviously had better scores in the initial 2010 survey so started from a much stronger position. In other cases, the DHB may have had a more concerted strategy for implementing clinical governance in place, in turn leading to survey respondent perceptions of better performance. In 2012, we conducted in-depth case studies of each DHB in parallel with the survey study through a mix of DHB self-reviews, document analysis and key informant interviews. Our aim was to analyse each DHB’s clinical governance strategy, the structures to facilitate clinical governance and leadership that had been implemented, and to learn about the challenges the DHB faced in the clinical governance development process [23]. As a general rule of thumb, the 2012 CGDI scores reported in this article matched closely the levels of development in each DHB and interviewee perceptions of performance. Thus, perhaps the most compelling explanation for the CGDI changes lies in where, in reality, on the trajectory of change the DHB was: some were clearly further along in their developmental journey, or had made considerably more progress in a short time than others, or had started to see the payoff from an early commitment to clinical governance. In some DHBs, it may be that there was simply more acceptance and knowledge of key concepts and terminology. In 2010 and 2012, we asked the question: ‘Clinical leadership is described as “…a new obligation to step up, work with other leaders, both clinical and managerial, and change the system where it would benefit patients”. How familiar are you with this concept?’ Some 51% of responding specialists expressed familiarity in 2010, [15] compared with 58% in 2012 [23]. Beyond the individual DHB setting, there had been no substantial changes to national policy settings between 2010 and 2012 so explanations for the CGDI changes would appear to lie in activities at the individual DHB level rather than the broader health system level, in contrast with explanations often provided in other policy studies [24,25].

While there was an overall CGDI score increase between 2010 and 2012, analysis of changes in the scores for individual index items is revealing with 5 of the 6 showing marked improvement. Survey respondents are obviously seeing development of partnership governance and decision making structures, increased attention to quality and safety in terms of both organisational resourcing as well as in care delivery, and increased clinical team responsibility for decision making. The item probing respondent participation in organisational design processes, on which improvement was not shown to be statistically significant, indicates that this is an area the DHBs perhaps need to focus more upon.

While our follow-up study does imply positive improvement, it also suggests on-going effort is required if New Zealand is to develop robust clinical governance across all DHBs. Some are obviously making more progress than others; some have made limited, if any, progress. Indeed, it might be suggested that the 2012 performance, as a whole, remains mediocre at 54% with the top scoring DHB achieving only 64%. The individual CGDI items, which link into both New Zealand government policy as well as literature on health system improvement, collectively provide pointers for where efforts might be directed. As noted in the introduction, and probed in these survey items, studies variously show that a strong focus on clinical leadership, quality and safety and teamwork can improve health system performance and patient outcomes while reducing costs [26–28]. Thus, the DHBs would do well to intensify their efforts in these areas.

Yet New Zealand, with its perhaps middling performance on clinical governance, is not alone. Elsewhere, studies show the struggle continues with attempting to bridge the managerial-clinical divide and build entities with strong clinical leadership focused on quality and safety [2,29,30]. The reasons for this may, again, be due to leadership capacity, as well as the context-dependent nature of any management and organisational development project which means it is difficult to simply transplant a well-functioning model of clinical governance from one organisation into another. Organisational re-development also takes considerable time, meaning higher CGDI scores could be envisaged as the developmental and implementation processes mature. The reason for New Zealand’s middling performance may also be due to health professionals, particularly senior doctors, having enough time in their already busy schedules to dedicate to clinical governance and leadership activities. They may also have limited interest in the ‘higher calling’ of leadership, especially if they have received limited training in the field and perhaps see this as a non-core duty. While our survey study did not probe this area, there is a strong argument that leadership training should feature early in health professional education and be promoted more widely [31–33]. This was a common theme in site visits and interviews with health professionals which we conducted in association with the 2012 survey, reported on elsewhere [23].

The research reported in this article has some limitations. First, the self-completed survey method relies on individual perceptions and has associated biases. However, response patterns were consistent across returned surveys from the different districts in both the 2010 and 2012 studies. Second, response rates above 52 and 32% for the two surveys respectively would have been desirable, but these rates were around the level obtained in comparable studies and there is a growing recognition that higher response rates are increasingly difficult to attain [34]. A review of research in a related field concluded that 55% is considered to be ‘one of the best response rates’ [35]. The low response rates were compounded by missing responses in the CGDI items, meaning that the 2012 results were ultimately based on responses from 21% of senior doctors. Very importantly, as noted, our respondent characteristics were similar to those of the broader health workforce suggesting that response bias would be limited. While we do not know whether non-responders had different perspectives from those who did respond, feedback received at presentations to New Zealand medical specialist conferences gives us no reason to believe a higher participation rate would have substantially changed the findings. That said, it is possible that the non-responders featured a larger proportion of professionals who felt un-engaged. If so, then the results in our study could indicate a higher level of clinical governance development than the reality. Third, our survey included only medical specialists. A follow-up study will permit tracking progress with clinical governance development from the perspective of the full range of New Zealand health professionals, given their participation in the 2012 survey. Fourth, the simple survey method means we were unable to probe why some DHBs performed better than others, or why some had achieved substantial changes between the two surveys. Answers to these questions might be explored through in-depth research methods such as reviews of policy documents and interviews with key informants. Finally, it would be useful to look at whether CGDI performances are predictive of performances on other measures in areas such as finance, organisational efficiency and patient safety.

## Conclusions

The CGDI and its application in the New Zealand context provides a baseline for further research. This might involve additional work on validating the questionnaire and CGDI, and adapting it for use in other health systems. It could also continue to be used for monitoring progress with clinical governance development in New Zealand. Of course, following from the above, a key question that arises is whether the New Zealand case is substantially different from that of other countries and, in turn, how applicable the CGDI is for use elsewhere. As noted in the introduction, New Zealand’s clinical governance policy drew heavily from the United Kingdom’s NHS; New Zealand’s health system is not dissimilar from other government-funded systems. Where New Zealand’s experience does differ from some countries that have pursued clinical governance could be in the relatively directive nature of policy emanating from central government, and in its smallness. Indeed, in a country such as North America, clinical governance is mostly an individual hospital-specific policy [36,37]. Yet this does not mean the CGDI could not be used in quite different health systems. In our study, we measured both local and national progress with clinical governance development through a lens predominantly that of specialists employed in individual hospitals. This implies that the index could be used in individual hospitals elsewhere, within a differently-structured health system, or to measure and compare performance more broadly in a system with characteristics that are closer to New Zealand’s.

## Abbreviations

ASMS: Association of Salaried Medical Specialists
CGCI: Clinical Governance Development Index
DHB: District Health Board
NHS: National Health Service
